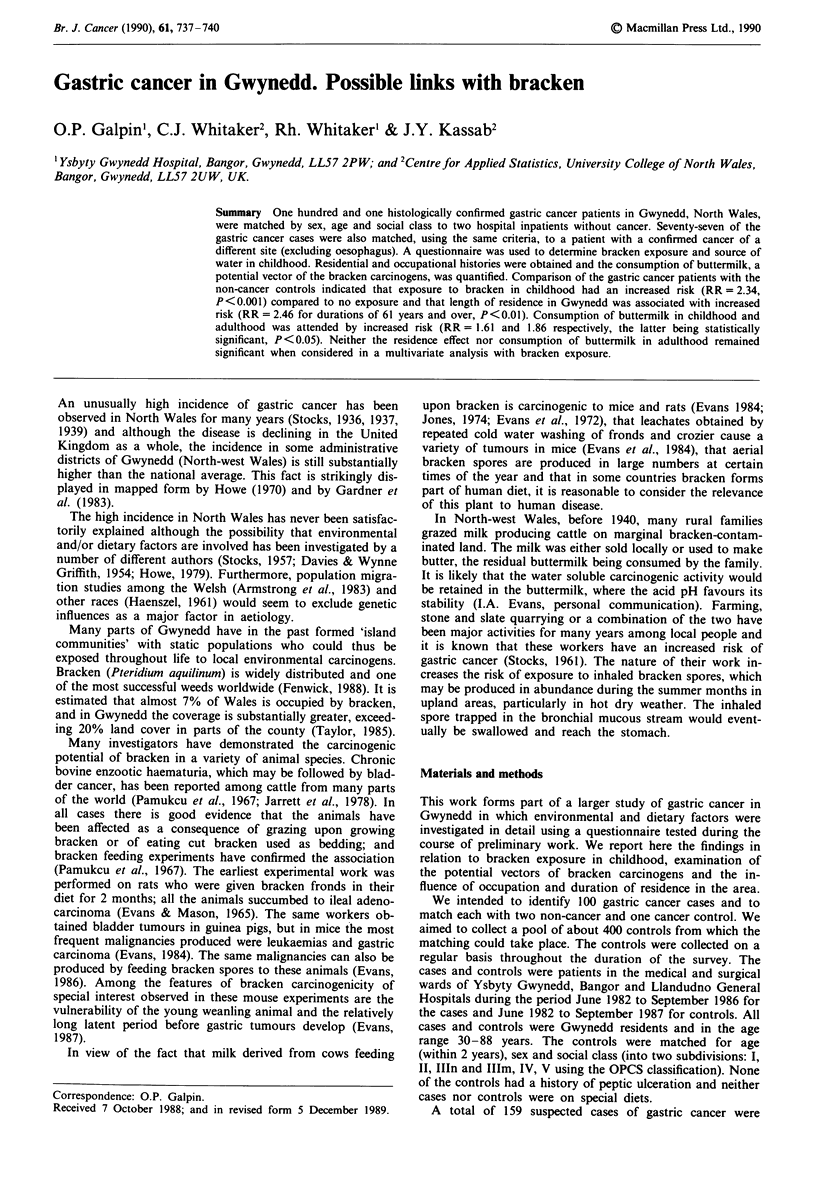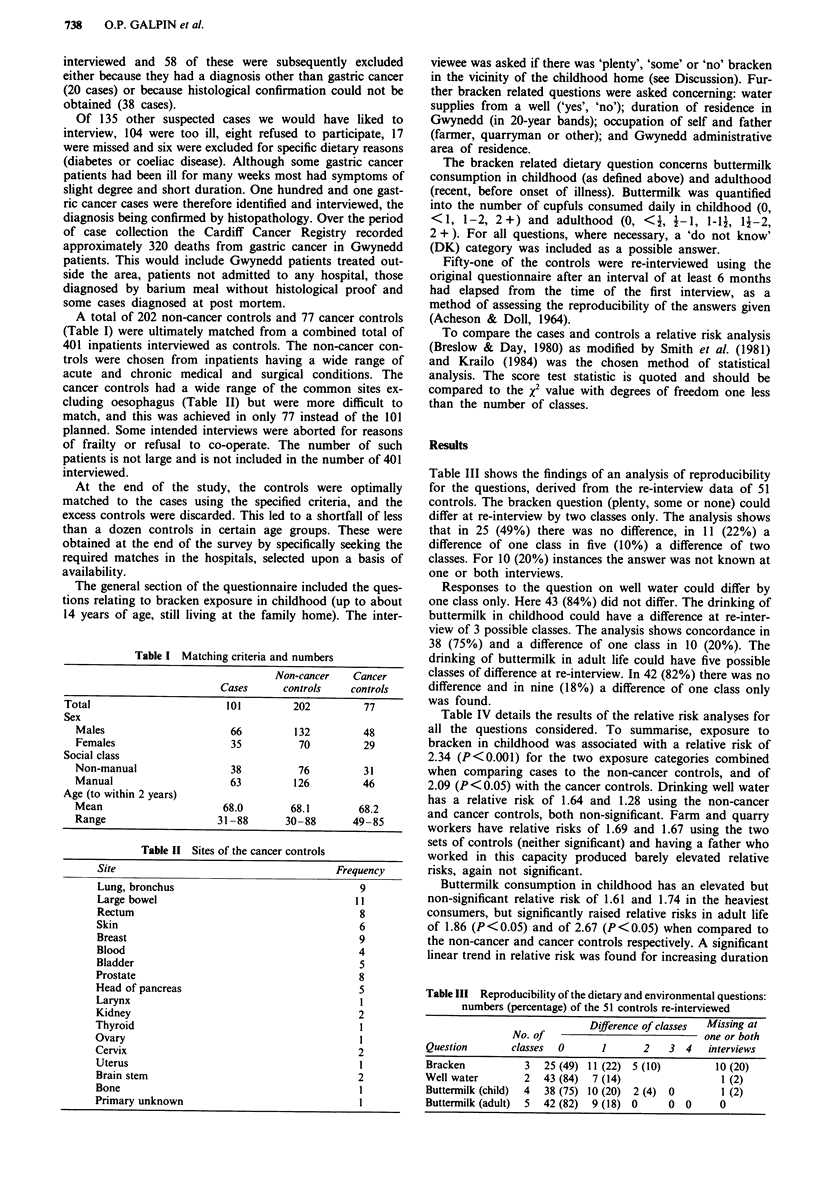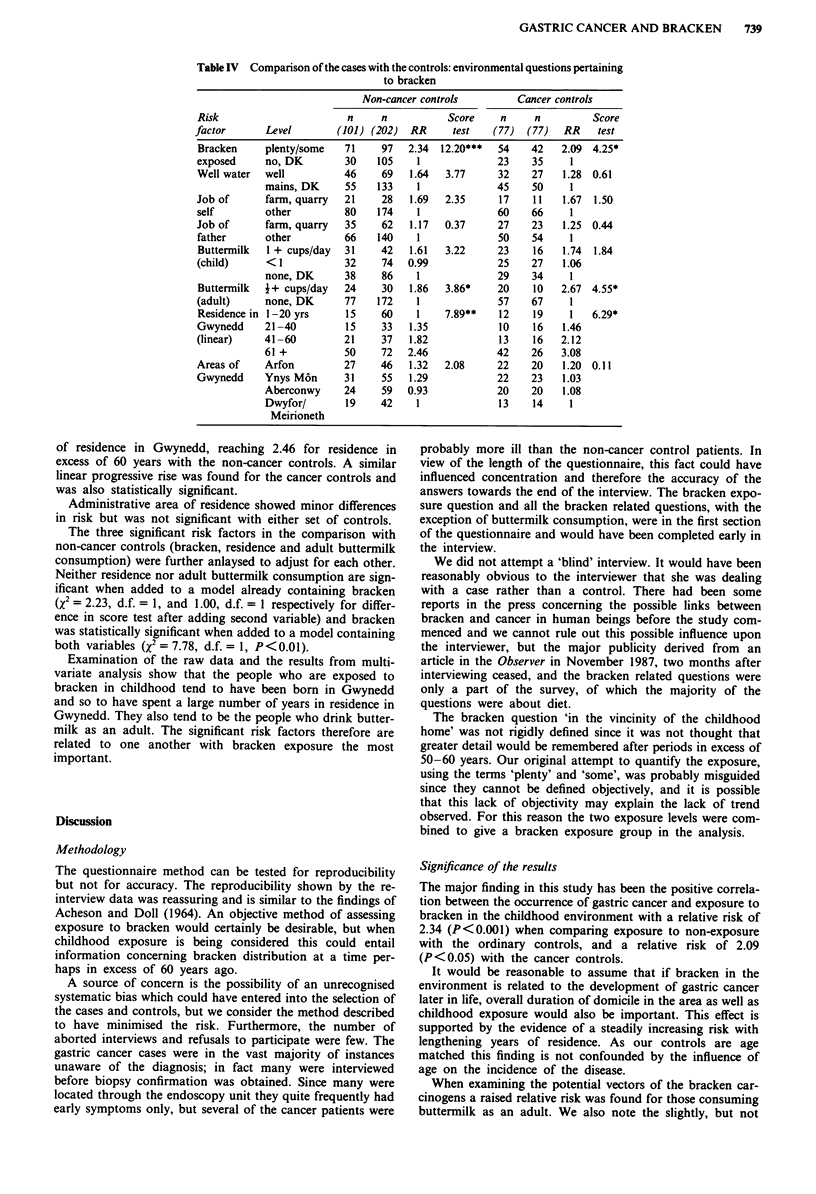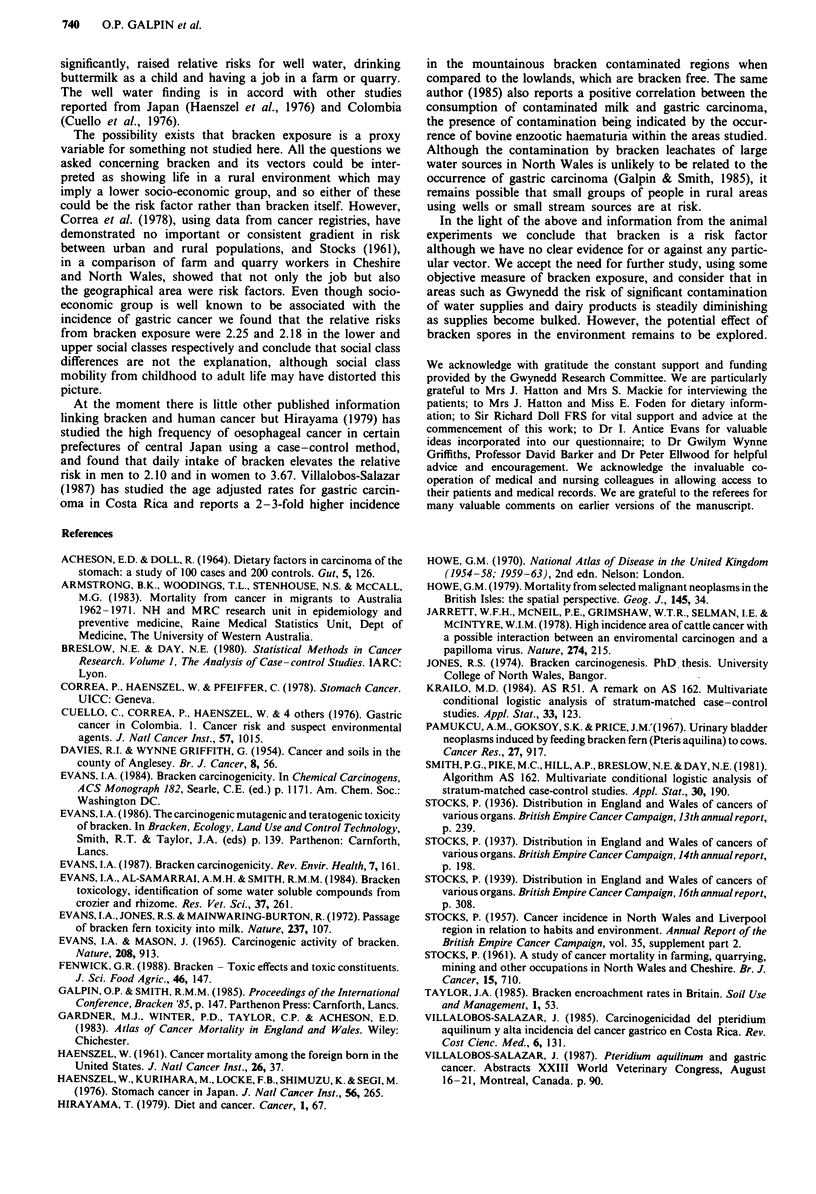# Gastric cancer in Gwynedd. Possible links with bracken.

**DOI:** 10.1038/bjc.1990.165

**Published:** 1990-05

**Authors:** O. P. Galpin, C. J. Whitaker, R. Whitaker, J. Y. Kassab

**Affiliations:** Ysbyty Gwynedd Hospital, Bangor.

## Abstract

One hundred and one histologically confirmed gastric cancer patients in Gwynedd, North Wales, were matched by sex, age and social class to two hospital inpatients without cancer. Seventy-seven of the gastric cancer cases were also matched, using the same criteria, to a patient with a confirmed cancer of a different site (excluding oesophagus). A questionnaire was used to determine bracken exposure and source of water in childhood. Residential and occupational histories were obtained and the consumption of buttermilk, a potential vector of the bracken carcinogens, was quantified. Comparison of the gastric cancer patients with the non-cancer controls indicated that exposure to bracken in childhood had an increased risk (RR = 2.34, P less than 0.001) compared to no exposure and that length of residence in Gwynedd was associated with increased risk (RR = 2.46 for durations of 61 years and over, P less than 0.01). Consumption of buttermilk in childhood and adulthood was attended by increased risk (RR = 1.61 and 1.86 respectively, the latter being statistically significant, P less than 0.05). Neither the residence effect nor consumption of buttermilk in adulthood remained significant when considered in a multivariate analysis with bracken exposure.


*Br. J. Cancer* (1990), **61**, 737–740 © Macmillan Press Ltd., 1990

### Gastric cancer in Gwynedd. Possible links with bracken

O.P. Galpin[1], C.J. Whitaker[2], Rh. Whitaker[1] & J.Y. Kassab[2]

[1]*Ysbyty Gwynedd Hospital, Bangor, Gwynedd, LL57 2PW; and* [2]*Centre for Applied Statistics, University College of North Wales, Bangor, Gwynedd, LL57 2UW, UK.*

**Summary** One hundred and one histologically confirmed gastric cancer patients in Gwynedd, North Wales, were matched by sex, age and social class to two hospital inpatients without cancer. Seventy-seven of the gastric cancer cases were also matched, using the same criteria, to a patient with a confirmed cancer of a different site (excluding oesophagus). A questionnaire was used to determine bracken exposure and source of water in childhood. Residential and occupational histories were obtained and the consumption of buttermilk, a potential vector of the bracken carcinogens, was quantified. Comparison of the gastric cancer patients with the non-cancer controls indicated that exposure to bracken in childhood had an increased risk (RR = 2.34, $P < 0.001$) compared to no exposure and that length of residence in Gwynedd was associated with increased risk (RR = 2.46 for durations of 61 years and over, $P < 0.01$). Consumption of buttermilk in childhood and adulthood was attended by increased risk (RR = 1.61 and 1.86 respectively, the latter being statistically significant, $P < 0.05$). Neither the residence effect nor consumption of buttermilk in adulthood remained significant when considered in a multivariate analysis with bracken exposure.

An unusually high incidence of gastric cancer has been observed in North Wales for many years (Stocks, 1936, 1937, 1939) and although the disease is declining in the United Kingdom as a whole, the incidence in some administrative districts of Gwynedd (North-west Wales) is still substantially higher than the national average. This fact is strikingly displayed in mapped form by Howe (1970) and by Gardner *et al.* (1983).

The high incidence in North Wales has never been satisfactorily explained although the possibility that environmental and/or dietary factors are involved has been investigated by a number of different authors (Stocks, 1957; Davies & Wynne Griffith, 1954; Howe, 1979). Furthermore, population migration studies among the Welsh (Armstrong *et al.*, 1983) and other races (Haenszel, 1961) would seem to exclude genetic influences as a major factor in aetiology.

Many parts of Gwynedd have in the past formed 'island communities' with static populations who could thus be exposed throughout life to local environmental carcinogens. Bracken (*Pteridium aquilinum*) is widely distributed and one of the most successful weeds worldwide (Fenwick, 1988). It is estimated that almost 7% of Wales is occupied by bracken, and in Gwynedd the coverage is substantially greater, exceeding 20% land cover in parts of the county (Taylor, 1985).

Many investigators have demonstrated the carcinogenic potential of bracken in a variety of animal species. Chronic bovine enzootic haematuria, which may be followed by bladder cancer, has been reported among cattle from many parts of the world (Pamukcu *et al.*, 1967; Jarrett *et al.*, 1978). In all cases there is good evidence that the animals have been affected as a consequence of grazing upon growing bracken or of eating cut bracken used as bedding; and bracken feeding experiments have confirmed the association (Pamukcu *et al.*, 1967). The earliest experimental work was performed on rats who were given bracken fronds in their diet for 2 months; all the animals succumbed to ileal adenocarcinoma (Evans & Mason, 1965). The same workers obtained bladder tumours in guinea pigs, but in mice the most frequent malignancies produced were leukaemias and gastric carcinoma (Evans, 1984). The same malignancies can also be produced by feeding bracken spores to these animals (Evans, 1986). Among the features of bracken carcinogenicity of special interest observed in these mouse experiments are the vulnerability of the young weanling animal and the relatively long latent period before gastric tumours develop (Evans, 1987).

In view of the fact that milk derived from cows feeding upon bracken is carcinogenic to mice and rats (Evans 1984; Jones, 1974; Evans *et al.*, 1972), that leachates obtained by repeated cold water washing of fronds and crozier cause a variety of tumours in mice (Evans *et al.*, 1984), that aerial bracken spores are produced in large numbers at certain times of the year and that in some countries bracken forms part of human diet, it is reasonable to consider the relevance of this plant to human disease.

In North-west Wales, before 1940, many rural families grazed milk producing cattle on marginal bracken-contaminated land. The milk was either sold locally or used to make butter, the residual buttermilk being consumed by the family. It is likely that the water soluble carcinogenic activity would be retained in the buttermilk, where the acid pH favours its stability (I.A. Evans, personal communication). Farming, stone and slate quarrying or a combination of the two have been major activities for many years among local people and it is known that these workers have an increased risk of gastric cancer (Stocks, 1961). The nature of their work increases the risk of exposure to inhaled bracken spores, which may be produced in abundance during the summer months in upland areas, particularly in hot dry weather. The inhaled spore trapped in the bronchial mucous stream would eventually be swallowed and reach the stomach.

### Materials and methods

This work forms part of a larger study of gastric cancer in Gwynedd in which environmental and dietary factors were investigated in detail using a questionnaire tested during the course of preliminary work. We report here the findings in relation to bracken exposure in childhood, examination of the potential vectors of bracken carcinogens and the influence of occupation and duration of residence in the area.

We intended to identify 100 gastric cancer cases and to match each with two non-cancer and one cancer control. We aimed to collect a pool of about 400 controls from which the matching could take place. The controls were collected on a regular basis throughout the duration of the survey. The cases and controls were patients in the medical and surgical wards of Ysbyty Gwynedd, Bangor and Llandudno General Hospitals during the period June 1982 to September 1986 for the cases and June 1982 to September 1987 for controls. All cases and controls were Gwynedd residents and in the age range 30–88 years. The controls were matched for age (within 2 years), sex and social class (into two subdivisions: I, II, IIIn and IIIm, IV, V using the OPCS classification). None of the controls had a history of peptic ulceration and neither cases nor controls were on special diets.

A total of 159 suspected cases of gastric cancer were

Correspondence: O.P. Galpin.
Received 7 October 1988; and in revised form 5 December 1989.

interviewed and 58 of these were subsequently excluded either because they had a diagnosis other than gastric cancer (20 cases) or because histological confirmation could not be obtained (38 cases).

Of 135 other suspected cases we would have liked to interview, 104 were too ill, eight refused to participate, 17 were missed and six were excluded for specific dietary reasons (diabetes or coeliac disease). Although some gastric cancer patients had been ill for many weeks most had symptoms of slight degree and short duration. One hundred and one gastric cancer cases were therefore identified and interviewed, the diagnosis being confirmed by histopathology. Over the period of case collection the Cardiff Cancer Registry recorded approximately 320 deaths from gastric cancer in Gwynedd patients. This would include Gwynedd patients treated outside the area, patients not admitted to any hospital, those diagnosed by barium meal without histological proof and some cases diagnosed at post mortem.

A total of 202 non-cancer controls and 77 cancer controls (Table I) were ultimately matched from a combined total of 401 inpatients interviewed as controls. The non-cancer controls were chosen from inpatients having a wide range of acute and chronic medical and surgical conditions. The cancer controls had a wide range of the common sites excluding oesophagus (Table II) but were more difficult to match, and this was achieved in only 77 instead of the 101 planned. Some intended interviews were aborted for reasons of frailty or refusal to co-operate. The number of such patients is not large and is not included in the number of 401 interviewed.

At the end of the study, the controls were optimally matched to the cases using the specified criteria, and the excess controls were discarded. This led to a shortfall of less than a dozen controls in certain age groups. These were obtained at the end of the survey by specifically seeking the required matches in the hospitals, selected upon a basis of availability.

The general section of the questionnaire included the questions relating to bracken exposure in childhood (up to about 14 years of age, still living at the family home). The interviewee was asked if there was 'plenty', 'some' or 'no' bracken in the vicinity of the childhood home (see Discussion). Further bracken related questions were asked concerning: water supplies from a well ('yes', 'no'); duration of residence in Gwynedd (in 20-year bands); occupation of self and father (farmer, quarryman or other); and Gwynedd administrative area of residence.

The bracken related dietary question concerns buttermilk consumption in childhood (as defined above) and adulthood (recent, before onset of illness). Buttermilk was quantified into the number of cupfuls consumed daily in childhood (0, $<1$, 1–2, 2+) and adulthood (0, $<\frac{1}{2}$, $\frac{1}{2}$–1, 1-$1\frac{1}{2}$, $1\frac{1}{2}$–2, 2+). For all questions, where necessary, a 'do not know' (DK) category was included as a possible answer.

Fifty-one of the controls were re-interviewed using the original questionnaire after an interval of at least 6 months had elapsed from the time of the first interview, as a method of assessing the reproducibility of the answers given (Acheson & Doll, 1964).

To compare the cases and controls a relative risk analysis (Breslow & Day, 1980) as modified by Smith *et al.* (1981) and Krailo (1984) was the chosen method of statistical analysis. The score test statistic is quoted and should be compared to the $\chi^2$ value with degrees of freedom one less than the number of classes.

**Table I** Matching criteria and numbers

| | *Cases* | *Non-cancer controls* | *Cancer controls* |
|---|---|---|---|
| Total | 101 | 202 | 77 |
| Sex | | | |
| Males | 66 | 132 | 48 |
| Females | 35 | 70 | 29 |
| Social class | | | |
| Non-manual | 38 | 76 | 31 |
| Manual | 63 | 126 | 46 |
| Age (to within 2 years) | | | |
| Mean | 68.0 | 68.1 | 68.2 |
| Range | 31–88 | 30–88 | 49–85 |

**Table II** Sites of the cancer controls

| *Site* | *Frequency* |
|---|---|
| Lung, bronchus | 9 |
| Large bowel | 11 |
| Rectum | 8 |
| Skin | 6 |
| Breast | 9 |
| Blood | 4 |
| Bladder | 5 |
| Prostate | 8 |
| Head of pancreas | 5 |
| Larynx | 1 |
| Kidney | 2 |
| Thyroid | 1 |
| Ovary | 1 |
| Cervix | 2 |
| Uterus | 1 |
| Brain stem | 2 |
| Bone | 1 |
| Primary unknown | 1 |

### Results

Table III shows the findings of an analysis of reproducibility for the questions, derived from the re-interview data of 51 controls. The bracken question (plenty, some or none) could differ at re-interview by two classes only. The analysis shows that in 25 (49%) there was no difference, in 11 (22%) a difference of one class in five (10%) a difference of two classes. For 10 (20%) instances the answer was not known at one or both interviews.

Responses to the question on well water could differ by one class only. Here 43 (84%) did not differ. The drinking of buttermilk in childhood could have a difference at re-interview of 3 possible classes. The analysis shows concordance in 38 (75%) and a difference of one class in 10 (20%). The drinking of buttermilk in adult life could have five possible classes of difference at re-interview. In 42 (82%) there was no difference and in nine (18%) a difference of one class only was found.

Table IV details the results of the relative risk analyses for all the questions considered. To summarise, exposure to bracken in childhood was associated with a relative risk of 2.34 ($P<0.001$) for the two exposure categories combined when comparing cases to the non-cancer controls, and of 2.09 ($P<0.05$) with the cancer controls. Drinking well water has a relative risk of 1.64 and 1.28 using the non-cancer and cancer controls, both non-significant. Farm and quarry workers have relative risks of 1.69 and 1.67 using the two sets of controls (neither significant) and having a father who worked in this capacity produced barely elevated relative risks, again not significant.

Buttermilk consumption in childhood has an elevated but non-significant relative risk of 1.61 and 1.74 in the heaviest consumers, but significantly raised relative risks in adult life of 1.86 ($P<0.05$) and of 2.67 ($P<0.05$) when compared to the non-cancer and cancer controls respectively. A significant linear trend in relative risk was found for increasing duration

**Table III** Reproducibility of the dietary and environmental questions: numbers (percentage) of the 51 controls re-interviewed

| *Question* | *No. of classes* | *Difference of classes* 0 | 1 | 2 | 3 | 4 | *Missing at one or both interviews* |
|---|---|---|---|---|---|---|---|
| Bracken | 3 | 25 (49) | 11 (22) | 5 (10) | | | 10 (20) |
| Well water | 2 | 43 (84) | 7 (14) | | | | 1 (2) |
| Buttermilk (child) | 4 | 38 (75) | 10 (20) | 2 (4) | 0 | | 1 (2) |
| Buttermilk (adult) | 5 | 42 (82) | 9 (18) | 0 | 0 | 0 | 0 |

**Table IV** Comparison of the cases with the controls: environmental questions pertaining to bracken

| | | *Non-cancer controls* | | | | *Cancer controls* | | | |
|---|---|---|---|---|---|---|---|---|---|
| *Risk factor* | *Level* | *n (101)* | *n (202)* | *RR* | *Score test* | *n (77)* | *n (77)* | *RR* | *Score test* |
| Bracken | plenty/some | 71 | 97 | 2.34 | 12.20*** | 54 | 42 | 2.09 | 4.25* |
| exposed | no, DK | 30 | 105 | 1 | | 23 | 35 | 1 | |
| Well water | well | 46 | 69 | 1.64 | 3.77 | 32 | 27 | 1.28 | 0.61 |
| | mains, DK | 55 | 133 | 1 | | 45 | 50 | 1 | |
| Job of | farm, quarry | 21 | 28 | 1.69 | 2.35 | 17 | 11 | 1.67 | 1.50 |
| self | other | 80 | 174 | 1 | | 60 | 66 | 1 | |
| Job of | farm, quarry | 35 | 62 | 1.17 | 0.37 | 27 | 23 | 1.25 | 0.44 |
| father | other | 66 | 140 | 1 | | 50 | 54 | 1 | |
| Buttermilk | 1 + cups/day | 31 | 42 | 1.61 | 3.22 | 23 | 16 | 1.74 | 1.84 |
| (child) | <1 | 32 | 74 | 0.99 | | 25 | 27 | 1.06 | |
| | none, DK | 38 | 86 | 1 | | 29 | 34 | 1 | |
| Buttermilk | ½+ cups/day | 24 | 30 | 1.86 | 3.86* | 20 | 10 | 2.67 | 4.55* |
| (adult) | none, DK | 77 | 172 | 1 | | 57 | 67 | 1 | |
| Residence in | 1–20 yrs | 15 | 60 | 1 | 7.89** | 12 | 19 | 1 | 6.29* |
| Gwynedd | 21–40 | 15 | 33 | 1.35 | | 10 | 16 | 1.46 | |
| (linear) | 41–60 | 21 | 37 | 1.82 | | 13 | 16 | 2.12 | |
| | 61 + | 50 | 72 | 2.46 | | 42 | 26 | 3.08 | |
| Areas of | Arfon | 27 | 46 | 1.32 | 2.08 | 22 | 20 | 1.20 | 0.11 |
| Gwynedd | Ynys Môn | 31 | 55 | 1.29 | | 22 | 23 | 1.03 | |
| | Aberconwy | 24 | 59 | 0.93 | | 20 | 20 | 1.08 | |
| | Dwyfor/ Meirioneth | 19 | 42 | 1 | | 13 | 14 | 1 | |

of residence in Gwynedd, reaching 2.46 for residence in excess of 60 years with the non-cancer controls. A similar linear progressive rise was found for the cancer controls and was also statistically significant.

Administrative area of residence showed minor differences in risk but was not significant with either set of controls.

The three significant risk factors in the comparison with non-cancer controls (bracken, residence and adult buttermilk consumption) were further anlaysed to adjust for each other. Neither residence nor adult buttermilk consumption are significant when added to a model already containing bracken ($\chi^2 = 2.23$, d.f. = 1, and 1.00, d.f. = 1 respectively for difference in score test after adding second variable) and bracken was statistically significant when added to a model containing both variables ($\chi^2 = 7.78$, d.f. = 1, $P < 0.01$).

Examination of the raw data and the results from multivariate analysis show that the people who are exposed to bracken in childhood tend to have been born in Gwynedd and so to have spent a large number of years in residence in Gwynedd. They also tend to be the people who drink buttermilk as an adult. The significant risk factors therefore are related to one another with bracken exposure the most important.

### Discussion

### *Methodology*

The questionnaire method can be tested for reproducibility but not for accuracy. The reproducibility shown by the re-interview data was reassuring and is similar to the findings of Acheson and Doll (1964). An objective method of assessing exposure to bracken would certainly be desirable, but when childhood exposure is being considered this could entail information concerning bracken distribution at a time perhaps in excess of 60 years ago.

A source of concern is the possibility of an unrecognised systematic bias which could have entered into the selection of the cases and controls, but we consider the method described to have minimised the risk. Furthermore, the number of aborted interviews and refusals to participate were few. The gastric cancer cases were in the vast majority of instances unaware of the diagnosis; in fact many were interviewed before biopsy confirmation was obtained. Since many were located through the endoscopy unit they quite frequently had early symptoms only, but several of the cancer patients were probably more ill than the non-cancer control patients. In view of the length of the questionnaire, this fact could have influenced concentration and therefore the accuracy of the answers towards the end of the interview. The bracken exposure question and all the bracken related questions, with the exception of buttermilk consumption, were in the first section of the questionnaire and would have been completed early in the interview.

We did not attempt a 'blind' interview. It would have been reasonably obvious to the interviewer that she was dealing with a case rather than a control. There had been some reports in the press concerning the possible links between bracken and cancer in human beings before the study commenced and we cannot rule out this possible influence upon the interviewer, but the major publicity derived from an article in the *Observer* in November 1987, two months after interviewing ceased, and the bracken related questions were only a part of the survey, of which the majority of the questions were about diet.

The bracken question 'in the vicinity of the childhood home' was not rigidly defined since it was not thought that greater detail would be remembered after periods in excess of 50–60 years. Our original attempt to quantify the exposure, using the terms 'plenty' and 'some', was probably misguided since they cannot be defined objectively, and it is possible that this lack of objectivity may explain the lack of trend observed. For this reason the two exposure levels were combined to give a bracken exposure group in the analysis.

### *Significance of the results*

The major finding in this study has been the positive correlation between the occurrence of gastric cancer and exposure to bracken in the childhood environment with a relative risk of 2.34 ($P < 0.001$) when comparing exposure to non-exposure with the ordinary controls, and a relative risk of 2.09 ($P < 0.05$) with the cancer controls.

It would be reasonable to assume that if bracken in the environment is related to the development of gastric cancer later in life, overall duration of domicile in the area as well as childhood exposure would also be important. This effect is supported by the evidence of a steadily increasing risk with lengthening years of residence. As our controls are age matched this finding is not confounded by the influence of age on the incidence of the disease.

When examining the potential vectors of the bracken carcinogens a raised relative risk was found for those consuming buttermilk as an adult. We also note the slightly, but not significantly, raised relative risks for well water, drinking buttermilk as a child and having a job in a farm or quarry. The well water finding is in accord with other studies reported from Japan (Haenszel et al., 1976) and Colombia (Cuello et al., 1976).

The possibility exists that bracken exposure is a proxy variable for something not studied here. All the questions we asked concerning bracken and its vectors could be interpreted as showing life in a rural environment which may imply a lower socio-economic group, and so either of these could be the risk factor rather than bracken itself. However, Correa et al. (1978), using data from cancer registries, have demonstrated no important or consistent gradient in risk between urban and rural populations, and Stocks (1961), in a comparison of farm and quarry workers in Cheshire and North Wales, showed that not only the job but also the geographical area were risk factors. Even though socio-economic group is well known to be associated with the incidence of gastric cancer we found that the relative risks from bracken exposure were 2.25 and 2.18 in the lower and upper social classes respectively and conclude that social class differences are not the explanation, although social class mobility from childhood to adult life may have distorted this picture.

At the moment there is little other published information linking bracken and human cancer but Hirayama (1979) has studied the high frequency of oesophageal cancer in certain prefectures of central Japan using a case–control method, and found that daily intake of bracken elevates the relative risk in men to 2.10 and in women to 3.67. Villalobos-Salazar (1987) has studied the age adjusted rates for gastric carcinoma in Costa Rica and reports a 2–3-fold higher incidence in the mountainous bracken contaminated regions when compared to the lowlands, which are bracken free. The same author (1985) also reports a positive correlation between the consumption of contaminated milk and gastric carcinoma, the presence of contamination being indicated by the occurrence of bovine enzootic haematuria within the areas studied. Although the contamination by bracken leachates of large water sources in North Wales is unlikely to be related to the occurrence of gastric carcinoma (Galpin & Smith, 1985), it remains possible that small groups of people in rural areas using wells or small stream sources are at risk.

In the light of the above and information from the animal experiments we conclude that bracken is a risk factor although we have no clear evidence for or against any particular vector. We accept the need for further study, using some objective measure of bracken exposure, and consider that in areas such as Gwynedd the risk of significant contamination of water supplies and dairy products is steadily diminishing as supplies become bulked. However, the potential effect of bracken spores in the environment remains to be explored.

We acknowledge with gratitude the constant support and funding provided by the Gwynedd Research Committee. We are particularly grateful to Mrs J. Hatton and Mrs S. Mackie for interviewing the patients; to Mrs J. Hatton and Miss E. Foden for dietary information; to Sir Richard Doll FRS for vital support and advice at the commencement of this work; to Dr I. Antice Evans for valuable ideas incorporated into our questionnaire; to Dr Gwilym Wynne Griffiths, Professor David Barker and Dr Peter Ellwood for helpful advice and encouragement. We acknowledge the invaluable co-operation of medical and nursing colleagues in allowing access to their patients and medical records. We are grateful to the referees for many valuable comments on earlier versions of the manuscript.

### References

ACHESON, E.D. & DOLL, R. (1964). Dietary factors in carcinoma of the stomach: a study of 100 cases and 200 controls. *Gut*, **5**, 126.

ARMSTRONG, B.K., WOODINGS, T.L., STENHOUSE, N.S. & MCCALL, M.G. (1983). Mortality from cancer in migrants to Australia 1962–1971. NH and MRC research unit in epidemiology and preventive medicine, Raine Medical Statistics Unit, Dept of Medicine, The University of Western Australia.

BRESLOW, N.E. & DAY, N.E. (1980). *Statistical Methods in Cancer Research. Volume 1, The Analysis of Case–control Studies*. IARC: Lyon.

CORREA, P., HAENSZEL, W. & PFEIFFER, C. (1978). *Stomach Cancer*. UICC: Geneva.

CUELLO, C., CORREA, P., HAENSZEL, W. & 4 others (1976). Gastric cancer in Colombia. 1. Cancer risk and suspect environmental agents. *J. Natl Cancer Inst.*, **57**, 1015.

DAVIES, R.I. & WYNNE GRIFFITH, G. (1954). Cancer and soils in the county of Anglesey. *Br. J. Cancer*, **8**, 56.

EVANS, I.A. (1984). Bracken carcinogenicity. In *Chemical Carcinogens, ACS Monograph 182*, Searle, C.E. (ed.) p. 1171. Am. Chem. Soc.: Washington DC.

EVANS, I.A. (1986). The carcinogenic mutagenic and teratogenic toxicity of bracken. In *Bracken, Ecology, Land Use and Control Technology*, Smith, R.T. & Taylor, J.A. (eds) p. 139. Parthenon: Carnforth, Lancs.

EVANS, I.A. (1987). Bracken carcinogenicity. *Rev. Envir. Health*, **7**, 161.

EVANS, I.A., AL-SAMARRAI, A.M.H. & SMITH, R.M.M. (1984). Bracken toxicology, identification of some water soluble compounds from crozier and rhizome. *Res. Vet. Sci.*, **37**, 261.

EVANS, I.A., JONES, R.S. & MAINWARING-BURTON, R. (1972). Passage of bracken fern toxicity into milk. *Nature*, **237**, 107.

EVANS, I.A. & MASON, J. (1965). Carcinogenic activity of bracken. *Nature*, **208**, 913.

FENWICK, G.R. (1988). Bracken – Toxic effects and toxic constituents. *J. Sci. Food Agric.*, **46**, 147.

GALPIN, O.P. & SMITH, R.M.M. (1985). *Proceedings of the International Conference, Bracken '85*, p. 147. Parthenon Press: Carnforth, Lancs.

GARDNER, M.J., WINTER, P.D., TAYLOR, C.P. & ACHESON, E.D. (1983). *Atlas of Cancer Mortality in England and Wales*. Wiley: Chichester.

HAENSZEL, W. (1961). Cancer mortality among the foreign born in the United States. *J. Natl Cancer Inst.*, **26**, 37.

HAENSZEL, W., KURIHARA, M., LOCKE, F.B., SHIMUZU, K. & SEGI, M. (1976). Stomach cancer in Japan. *J. Natl Cancer Inst.*, **56**, 265.

HIRAYAMA, T. (1979). Diet and cancer. *Cancer*, **1**, 67.

HOWE, G.M. (1970). *National Atlas of Disease in the United Kingdom (1954–58; 1959–63)*, 2nd edn. Nelson: London.

HOWE, G.M. (1979). Mortality from selected malignant neoplasms in the British Isles: the spatial perspective. *Geog. J.*, **145**, 34.

JARRETT, W.F.H., MCNEIL, P.E., GRIMSHAW, W.T.R., SELMAN, I.E. & MCINTYRE, W.I.M. (1978). High incidence area of cattle cancer with a possible interaction between an enviromental carcinogen and a papilloma virus. *Nature*, **274**, 215.

JONES, R.S. (1974). Bracken carcinogenesis. PhD thesis. University College of North Wales, Bangor.

KRAILO, M.D. (1984). AS R51. A remark on AS 162. Multivariate conditional logistic analysis of stratum-matched case–control studies. *Appl. Stat.*, **33**, 123.

PAMUKCU, A.M., GOKSOY, S.K. & PRICE, J.M. (1967). Urinary bladder neoplasms induced by feeding bracken fern (Pteris aquilina) to cows. *Cancer Res.*, **27**, 917.

SMITH, P.G., PIKE, M.C., HILL, A.P., BRESLOW, N.E. & DAY, N.E. (1981). Algorithm AS 162. Multivariate conditional logistic analysis of stratum-matched case-control studies. *Appl. Stat.*, **30**, 190.

STOCKS, P. (1936). Distribution in England and Wales of cancers of various organs. *British Empire Cancer Campaign, 13th annual report*, p. 239.

STOCKS, P. (1937). Distribution in England and Wales of cancers of various organs. *British Empire Cancer Campaign, 14th annual report*, p. 198.

STOCKS, P. (1939). Distribution in England and Wales of cancers of various organs. *British Empire Cancer Campaign, 16th annual report*, p. 308.

STOCKS, P. (1957). Cancer incidence in North Wales and Liverpool region in relation to habits and environment. *Annual Report of the British Empire Cancer Campaign*, vol. 35, supplement part 2.

STOCKS, P. (1961). A study of cancer mortality in farming, quarrying, mining and other occupations in North Wales and Cheshire. *Br. J. Cancer*, **15**, 710.

TAYLOR, J.A. (1985). Bracken encroachment rates in Britain. *Soil Use and Management*, **1**, 53.

VILLALOBOS-SALAZAR, J. (1985). Carcinogenicidad del pteridium aquilinum y alta incidencia del cancer gastrico en Costa Rica. *Rev. Cost Cienc. Med.*, **6**, 131.

VILLALOBOS-SALAZAR, J. (1987). *Pteridium aquilinum* and gastric cancer. Abstracts XXIII World Veterinary Congress, August 16–21, Montreal, Canada. p. 90.